# MetaTropismDB: a database of organ-specific metastasis induced by human cancer cell lines in mouse models

**DOI:** 10.1093/database/baaa100

**Published:** 2020-11-25

**Authors:** Matteo Giulietti, Marco Bastianoni, Monia Cecati, Annamaria Ruzzo, Massimo Bracci, Marco Malavolta, Francesco Piacenza, Robertina Giacconi, Francesco Piva

**Affiliations:** Department of Specialistic Clinical and Odontostomatological Sciences, Polytechnic University of Marche, Via Brecce Bianche, 60131, Ancona, Italy; Department of Specialistic Clinical and Odontostomatological Sciences, Polytechnic University of Marche, Via Brecce Bianche, 60131, Ancona, Italy; Department of Specialistic Clinical and Odontostomatological Sciences, Polytechnic University of Marche, Via Brecce Bianche, 60131, Ancona, Italy; Department of Biomolecular Sciences, University of Urbino “Carlo Bo”, Via Sant’Andrea 34, 61029, Urbino, Italy; Occupational Medicine, Department of Clinical and Molecular Sciences, Polytechnic University of Marche, Via Tronto 10/a, 60126, Ancona, Italy; Advanced Technology Center for Aging Research, Scientific Technological Area, IRCCS INRCA, Via Birarelli 8, 60121, Ancona, Italy; Advanced Technology Center for Aging Research, Scientific Technological Area, IRCCS INRCA, Via Birarelli 8, 60121, Ancona, Italy; Advanced Technology Center for Aging Research, Scientific Technological Area, IRCCS INRCA, Via Birarelli 8, 60121, Ancona, Italy; Department of Specialistic Clinical and Odontostomatological Sciences, Polytechnic University of Marche, Via Brecce Bianche, 60131, Ancona, Italy

## Abstract

The organotropism is the propensity of metastatic cancer cells to colonize preferably certain distant organs, resulting in a non-random distribution of metastases. In order to shed light on this behaviour, several studies were performed by the injection of human cancer cell lines into immunocompromised mouse models. However, the information about these experiments is spread in the literature. For each xenograft experiment reported in the literature, we annotated both the experimental conditions and outcomes, including details on inoculated human cell lines, mouse models, injection methods, sites of metastasis, organs not colonized, rate of metastasis, latency time, overall survival and the involved genes. We created MetaTropismDB, a freely available database collecting hand-curated data useful to highlight the mechanisms of organ-specific metastasis. Currently, it stores the results of 513 experiments in which injections of 219 human cell lines have been carried out in mouse models. Notably, 296 genes involved in organotropic metastases have been collected. This specialized database allows the researchers to compare the current results about organotropism and plan future experiments in order to identify which tumour molecular signatures establish if and where the metastasis will develop.

**Database URL:**  http://www.introni.it/Metastasis/metastasis.html

## Introduction

Despite the recent therapeutic advances in cancer treatment, the development of metastasis is still the main cause of death. This process, leading to the spread of cancer cells from the primary tumour site to distant anatomical sites, consists of various phases. The phases encompass the local invasion of tumour cells into the adjacent tissue, the intravasation and extravasation phases in the circulatory system and the colonization of the target organs. In this new environment, the circulating tumour cells establish micrometastases, also supported by the primary tumour via secreted factors, including cytokines and extracellular vesicles (1).

An aspect of increasing interest about this exacerbation is the non-random distribution of metastases, which is different for each cancer type, known as organotropism (2). Indeed, cancer cells leaving the primary tumour site have the propensity to colonize preferably certain distant organs. For example, breast cancer patients almost exclusively show metastases in bones, lungs, brain and liver, whereas prostate cancer patients show metastases mainly in bones (3). Moreover, the luminal subtype of breast cancer has a greater propensity to metastasize to bones than the triple-negative subtype of breast cancer (4). Some metastasis patterns can be explained by the Ewing hypothesis, according to which circulatory patterns can direct cancer cells. However, solid evidence showed that organotropism is rather due to the molecular characteristics of both cancer cells and target sites, according to the ‘seed and soil’ hypothesis of Paget (1, 2).

The investigation about the mechanisms of metastasis formation and organotropism is generally performed using mouse models that mimic the various stages of cancer cell homing and spreading. This surrogate system for human organ-specific metastasis is based on the transplantation of human cancer cell lines in immunocompromised mice (5, 6). In particular, several mouse models carrying different mutations that lead to different levels of immunodeficiency, including BALB/c athymic nude, NOD SCID and NOD SCID gamma (NSG) mice, have been developed. In these models, cancer cells can be injected directly into the circulatory system (experimental metastasis assay) or into a mouse organ (spontaneous metastasis assay). While the experimental assay describes only the last steps of metastasis formation, i.e. cancer cell dissemination in blood and extravasation and colonization of the target organ, the spontaneous assays have the advantage of depicting also the early phases, i.e. local invasion and intravasation. Furthermore, the spontaneous metastasis assays can be classified into orthotopic, when cancer cells are implanted into the organ from which they derive, or ectopic, when other organs or tissues, mainly the skin, are involved. Subcutaneous transplantation causes a rapid growth of tumours that can be easily manipulated; instead, the orthotopic injection better recapitulate the tumorigenesis and the metastasis development since it allows the interactions with the tissue of origin (5, 6).

The distribution patterns of metastases induced by different cancer cell lines or their sublines, deriving from *in vivo* selection of clones with a particular tropism, allow the identification of gene expression signatures related to organotropism. For example, genes directing the metastatic breast cancer cell lines towards bone, lung or brain have been identified by xenograft experiments (7–9). Moreover, it is known that tumour-derived extracellular vesicles can condition the target organs to facilitate the attachment of circulating cancer cells. In this regard, the proteomic profiling of the exosomes released from breast and pancreatic cancer cell lines with different organotropism revealed that some integrins mediated the metastases in lung or liver (10). Interestingly, the pre-administration of tumour-derived extracellular vesicles with a different tropism can redirect the metastasis distribution, indicating a key role of these vesicles and their components in organotropism (10).

The experiments about organotropism assessed by the xenograft models are spread in the literature; therefore, there is a need to collect this information in a unique resource, allowing researchers to realize the current knowledge about organ-specific metastasis, compare the results and plan next experiments to highlight the molecular mechanisms underlying the metastatic cell seeding in specific sites. Here, by hand-curated literature analysis, we collected organotropism data from mouse xenograft trans-plantation studies of human cancer cell lines in MetaT-ropismDB database (http://www.introni.it/Metastasis/metastasis.html).

## Materials and methods

Literature analysis included all studies reporting the metastasis development in immunosuppressed mice following the injection of human cancer cell lines. Notably, we also annotated the experiments in which the metastasis development was not observed. For each paper, we collected both the adopted experimental procedures and the obtained results. In particular, we annotated the injected cell line along with its concentration and the medium at the moment of administration, the inoculation method, the visualization techniques to identify and localize the metastases and the adopted mouse models and their age. Moreover, we collected all the reported results, including primary tumour and metastasis sites, such as their latency, the frequency of mice that developed metastases, the overall survival times and the genes involved in organotropism. When the frequency of mice developing metastases was not reported, we anyway annotated the presence of metastases in the Notes field. We also assigned a fitting degree between the metastasis sites reported in each experiment and those observed in humans for the corresponding cancer type. We retrieved the frequencies of human metastasis sites from the literature (11–13). In particular, we assigned ‘0 (no fit)’ to experiments that do not induce metastasis in mice. We assigned score ‘3 (high)’ when at least one metastasis site developed in mice corresponds to the most frequent metastatic site in human for the same primary tumour. In addition, ‘1 (low)’ and ‘2 (medium)’ scores mean that metastasis sites observed in mice correspond to only one or more than one human metastatic sites, respectively, excluding the most frequent site.

For storage and query of the data, we constructed a MySQL relational database, organized in four tables. The main table stores data regarding each xenograft experiment, including both the experimental conditions and the obtained results. Each record of this table is linked to the records of the other three tables, collecting information about the human cell lines, the mouse models and the references, respectively. In the table of cell lines we collected their official name, a short description, the organ from which they derive, their origin (that is, if they derive from another cell line or were isolated from a patient) and links to various cell line databases, including Cellosaurus (https://web.expasy.org/cellosaurus/), DSMZ (www.dsmz.de), Cell Model Passports (https://cellmodelpassports.sanger.ac.uk) and CellFinder (www.cellfinder.org). The mouse model table contains the nomenclature of mice, including links to the main companies which develop and supply mouse models and to other databases collecting mouse genomic and phenotypic information, such as The Jackson Laboratory (JAX, www.jax.org), Charles River Laboratories Inc. (www.criver.com), Envigo (www.envigo.com), Janvier Labs (https://janvier-labs.com) and Taconic Biosciences (www.taconic.com). Moreover, the links to other dat-abases with mouse genomic and phenotypic informa-tion, such as Mouse Genome Informatics (www.informatics.jax.org) and Mouse Phenome Database (https://phenome.jax.org) are stored in this table. Finally, the fourth table stores the references of the articles from which data were extracted, including PubMed IDs.

**Figure 1.**
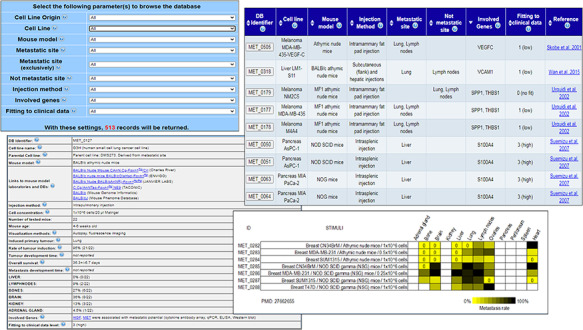
MetaTropismDB web interfaces. At the top, left: the Browse page which allows the selection of search parameters. At the top, right: search result page showing the synthetic information of each experiment. At the bottom, left: card with full details of each experiment. At the bottom, right: an image at the bottom of each card shows the metastasis patterns as a result of different experimental conditions assessed in the same study.

In order to facilitate the visualization and retrieval of the data, we also created a user-friendly web interface (Figure 1) by using Perl DBI/CGI, Javascript and Ajax/XML languages. MetaTropismDB supports all major browsers are supported. The web interface allows the users to browse the experiments or to search for a specific combination of cancer cell line, its tissue of origin, injection method, mouse model, site of metastasis, site of not metastasis, involved genes and fitting to clinical data.

## Results

### Current status of MetaTropismDB

The database MetaTropismDB collects organotropism data exclusively obtained from xenograft experiments of human cancer cell lines inoculated, by various techniques, in immunodeficient mouse models. Notably, the database also includes negative results, i.e. the absence of any type of metastasis as a result of inoculation of human cancer cell lines. Overall, it contains 513 records describing both the experimental conditions and the obtained results of each experiment. Since a single experiment usually contained frequency data of metastases in multiple organs, the overall number of metastatic sites collected is 746 and that of the sites without metastases is 404. According to the database data, the main colonized organs were bones, brain, liver, lungs and lymph nodes, whereas the other 31 target organs reported in this database are less frequently colonized. Currently, MetaTropismDB collects metastatic organotropism data of 219 human cancer cell lines, which mainly derive from breast, colorectal, lung, prostate, bladder, pancreatic, thyroid and renal cancers and melanoma. The official names of cell lines were reported according to Cellosaurus database (https://web.expasy.org/cellosaurus/) or to the literature. In MetaTropismDB database, 18 different mouse models are described, with the BALB/c athymic nude mice being the most frequently adopted model. Other immunocompromised mice collected in this database are the athymic nude, C.B-17 SCID beige, C57BL/6, MF1, NIH-III nude, NMRI nude, NSG, NOD SCID, NOG and Swiss athymic nude mice. Notably, also the nomenclature of these mouse models is defined according to the literature and to the companies which develop and supply the mouse models.

Regarding the experimental procedures used for the injection of human cancer cells into mice, the orthotopic one (i.e. injection in the organ of origin of the cell line) was the most frequent, with the subcutaneous, the intracardiac or tail vein injection being less adopted. Moreover, among the experimental methods, the most common one was the inoculation into the mouse tail vein. For the visualization and identification of metastases in murine organs, the most commonly adopted method was autopsy and subsequent histological examination; however, bioluminescence and fluorescence *in vivo* imaging are increasingly used.

The results of xenograft experiments collected in MetaTropismDB include the site of the induced primary tumour, its take rate, the colonized organs, the corresponding metastasis frequency and the development time. Finally, when the authors of the study identified the genes involved in the organotropism in their experimental model, we listed these genes and linked them to the NCBI Gene database. In particular, the association between a gene and a specific tropism is generally identified by assessing its expression by microarray, quantitative PCR or western blot in an organ-specific metastatic cell line. About 300 genes involved in organ-specific metastasis were collected in MetaTropismDB, along with the methods that allowed their identification.

### Using MetaTropismDB

The web interface allows the users to browse the experiments or to search (Figure 1) allows the users to freely download the entire database in different file formats, to browse the experiments or to search for a specific combination of tissue of origin of cell line, cell line name, injection method, mouse model, site of metastasis, non-metastasis site, involved genes and fitting to clinical data. The number of possible resulting records is shown dynamically. After the selection of criteria, a synthesis of the search results is shown in a new page and the user can select those of interest in order to retrieve the full description of each experiment with all the experimental conditions and the obtained results (Figure 1). Notably, many links to the relative entries in other relevant databases are provided for both the human cancer cell lines and the mouse models. The wide use of cross-links allows an easy recovery of specific information about the biological, genetic and phenotypic characteristics of human cancer cell lines and mouse models under investigation. Moreover, the direct links to the NCBI Gene database and PubMed are also provided. The card regarding an experiment includes also a Notes field that contains additional information, for example, the specific bone affected by metastasis, if mice have been humanized by human tissue implants, or the metastatic organs when neither the occurrence nor the frequency (out of the number of tested mice) were clearly reported in the paper.

At the bottom of this card, an image summarizes the effects of different experimental conditions assessed in the same study on organotropism. The results are reported through a yellow/black colour scale, with yellow meaning low frequency of metastases (if completely absent, a 0 is added) and black meaning 100% of mice with metastases. This image helps the users to easily realize how the different experimental conditions, which are comparable only within the same study, can affect the metastasis development and organotropism.

Finally, a detailed explanation of the search options and result fields is available at the Help page. We plan to continuously update MetaTropismDB, thanks to the suggestions of users.

### Genes involved in lung and bone metastases

By using MetaTropismDB, we selected all the experiments that assessed the metastasis formation exclusively in lungs or in bones of mice injected by human cancer cell lines. Then, the associated genes, collected in the respective records, have been submitted to Enrichr tool (14, https://amp.pharm.mssm.edu/Enrichr/) in order to perform enrichment analyses and highlight the diseases and organ-specific metastasis pathways (Supplementary Table S1). Regarding lung metastasis genes, the enrichment analysis identified the disease ‘Secondary malignant neoplasm of lung’ (*P* = 5.79e-23), thus confirming the involvement of these genes in lung metastasis. Moreover, we identified some interesting cellular pathways; among them, the RAGE- and osteopontin-mediated pathways are already known to be involved in lung-specific metastases from different primary tumours. In particular, Receptor for Advanced Glycation Endproducts overexpression in breast cancer cells promoted lung metastasis *in vivo* (15), while osteopontin increased the adhesion to lung cells and the metastasis of osteosarcoma cells (16). Regarding bone metastasis genes, the enrichment analysis, as expected, identified the ‘Secondary malignant neoplasm of bone’ disease (*P* = 5.04e-20). Moreover, the pathway libraries highlighted the CXCR4-mediated signalling as the most involved pathway (*P* = 4.77e-08). Indeed, it is known that the osteotropism of CXCR4 + cancer stem cells depends on their ability to follow the CXCL12 gradient towards the bone marrow (17, 18). Notably, the bone tropism can also be attained by melanoma-derived exosomes, which are able to induce the CXCL12/CXCR4 axis (19).

## Discussion

MetaTropismDB is the first resource that collects information about the organ-specific metastasis of human cancer cell lines engrafted in mouse models. Importantly, data about not colonized organs are also included, when experimentally validated. This database allows the researchers to easily highlight the human cell lines with similar metastatic organotropism or, alternatively, cell lines deriving from the same type of primary tumour but showing different organotropism. For example, commonly expressed genes among different cell lines targeting the same organ, or differentially expressed genes among similar cell lines targeting two different organs, could be investigated in order to identify the genes responsible for the target specificity. For example, as observed in this type of studies collected in our database, lung metastasis from bladder cancer has been shown to be mediated by CD24 (20), whereas ITGA1 was involved in lymph node metastasis of hepatocellular carcinoma (21). Furthermore, the fitting score to clinical data allows the user to realize the extent to which the experiments in mice reproduce the metastasis pattern in humans. For example, the breast cancer MDA-MB-231 cell line does not induce metastasis in mice (fitting score = 0), but its subline 1833 induces metastasis in bone (score = 3, since bone is the most frequent metastatic site of human breast cancer) and the subline 1834 induces metastasis in lungs (score = 1, since lungs are not the most frequent target tissue) of mice (9, 13).

This database also allows the conscious selection of the most suitable injection methods and murine models for the future xenograft experiments, since they can affect the pattern of metastasis induced by the same cancer cell line. For example, melanoma MDA-MB-435 and prostate PC-3 cancer cell lines formed bone metastasis only through intracardiac injection (22, 23), and pancreatic Panc-1 cancer cells formed liver metastasis only in NOG mice (24). In general, the orthotopic injection method should be preferred since it can depict also the local invasion in the tissue of origin and the interactions with tumour microenvironment, thus describing all phases of tumorigenesis and metastasis development (5, 6). Although the functional host immune system contributes to tumour and metastasis formation (25), its specific role in the immunocompromised mouse models cannot be assessed. In order to partially overcome this limitation, less immunocompromised (e.g. athymic nude mice) and humanized mouse models could be used (26, 27). Interestingly, among the visualization methods, the bioluminescence *in vivo* imaging is increasingly adopted, seems to be very accurate and comparable with histological examination and allows the tracking of the course of metastasis (20, 28).

## Conclusions

In conclusion, since the genes involved in organotropism could represent the new biomarkers of organ-specific metastasis and since the site of metastasis is a predictor of survival, for example in melanoma patients (29), these molecular signatures could improve the patient prognosis stratification. The investigations about organotropism are in progress and promise to identify the underlying molecular mechanisms that may suggest new therapeutic targets, allowing the development of preventive, organ-specific and personalized therapies (30). In this regard, it could be possible to perform treatments to silence the expression of genes responsible for the metastatic cell attachment in distal organs—for example, it could be possible by analysing the gene expression data repositories and identifying the conditions (food, lifestyle, chemicals, gene therapy, etc.) able to restore the expression of a gene of interest.

## Supplementary Material

baaa100_Supp
